# Screening Protocols for Group B Streptococcus: Are Transport Media Appropriate?

**DOI:** 10.1080/10647440300025521

**Published:** 2003

**Authors:** Nicolette Teese, Daneeta Henessey, Christopher Pearce, Nigel Kelly, Suzanne Garland

**Affiliations:** Department of Microbiology and Infectious Diseases The Royal Women's Hospital Women's and Children's Health Flemington Road, Parkville Victoria 3053 Australia

## Abstract

*Objective:* To evaluate group B streptococcus (GBS) detection in an *in vitro* setting, using a low and controlled inoculum from swabs directly inoculated into a selective medium, as compared to delayed inoculation following a period in a commercial Amies transport medium with charcoal (Venturi Transystem^™^ Copan, Italy).

*Study design:* Clinical isolates of GBS (*n* = 103), were inoculated into the Amies transport medium with charcoal in a concentration of 100 colony-forming units (cfu)/ml (10 cfu/swab). Swabs were then transferred to an enrichment broth (NPC) at time intervals of 0, 2, 4, 6 and 24 hours. Broths were then incubated for 18–24 hours at 35^°^C in air, before being transferred to New Granada Medium Modified (NGM) for GBS detection and incubated for a further 18–24 hours at 35^°^C in air. If the characteristic orange pigmented colonies were observed after this period, the specimen was recorded as + (1–10 colonies) or ++ (more than 10 colonies).

*Results:* Overall 92.2% (95/103) of isolates were detected in all tubes and at all times. An additional two isolates were non-hemolytic, non-pigment forming GBS. Of note, 3.9% (4/103) were negative until 2 hours delayed inoculation and 1.9% (2/103) gave inconsistent results, likely due to the low inoculum used.

*Conclusion:* Delayed inoculation into selective enrichment broth following a period in transport medium, even with a low inoculum, gave a similar and acceptable GBS detection rate to direct inoculation. Hence, Amies transport medium with charcoal is an appropriate transport medium to use, where it is not practical for clinical specimens to be directly inoculated into selective enrichment broth and as endorsed in the Centers for Diseases Control (CDC) Guidelines, 2002.


*Infect Dis Obstet Gynecol 2003;11:199–202*

### Screening protocols for group B streptococcus: are transport media appropriate?

**Nicolette Teese, Daneeta Henessey, Christopher Pearce, Nigel Kelly and Suzanne Garland**

*Department of Microbiology and Infectious Diseases, The Royal Women's Hospital, Women's and Children's Health, Flemington Road, Parkville, Victoria, Australia*

***Objective:*** To evaluate group B streptococcus (GBS) detection in an *in vitro* setting, using a low and controlled inoculum from swabs directly inoculated into a selective medium, as compared to delayed inoculation following a period in a commercial Amies transport medium with charcoal (Venturi Transystem™ Copan, Italy).
***Study design:*** Clinical isolates of GBS ($n = 103$), were inoculated into the Amies transport medium with charcoal in a concentration of 100 colony-forming units (cfu)/ml (10 cfu/swab). Swabs were then transferred to an enrichment broth (NPC) at time intervals of 0, 2, 4, 6 and 24 hours. Broths were then incubated for 18–24 hours at 35°C in air, before being transferred to New Granada Medium Modified (NGM) for GBS detection and incubated for a further 18–24 hours at 35°C in air. If the characteristic orange pigmented colonies were observed after this period, the specimen was recorded as + (1–10 colonies) or ++ (more than 10 colonies).
***Results:*** Overall 92.2% (95/103) of isolates were detected in all tubes and at all times. An additional two isolates were non-hemolytic, non-pigment forming GBS. Of note, 3.9% (4/103) were negative until 2 hours delayed inoculation and 1.9% (2/103) gave inconsistent results, likely due to the low inoculum used.
***Conclusion:*** Delayed inoculation into selective enrichment broth following a period in transport medium, even with a low inoculum, gave a similar and acceptable GBS detection rate to direct inoculation. Hence, Amies transport medium with charcoal is an appropriate transport medium to use, where it is not practical for clinical specimens to be directly inoculated into selective enrichment broth and as endorsed in the Centers for Diseases Control (CDC) Guidelines, 2002.

Key words: GROUP B STREPTOCOCCUS; SCREENING; TRANSPORT MEDIA; AMIES TRANSPORT MEDIUM WITH CHARCOAL (COPAN VENTURI TRANSYSTEM™)

Neonatal Group B streptococcus (GBS) sepsis remains a significant cause of morbidity and mortality[1,2]. Although the incidence of perinatal GBS disease has declined since the introduction of intrapartum antibiotic therapy[3], it still remains a leading cause of neonatal GBS disease[2]. In the prevention strategy of screening pregnant women for colonization, optimal detection of GBS is paramount[2–6].

To maximize detection rates it is advised that clinical swabs be taken from both the low vagina and anorectal region and placed directly into a GBS selective broth prior to inoculation onto a selective solid/semi-solid medium[2,5]. The revised

Correspondence to: Associate Professor Suzanne Garland, Director of Microbiological Research, Clinical Microbiology and Infectious Diseases, The Royal Women's Hospital, 132 Grattan Street, Carlton, Victoria 3053, Australia. Email: suzanne.garland@wch.org.au

© 2003 The Parthenon Publishing Group

CDC guidelines of 2002 recommend the use of a transport medium such as Amies[6]. To the best of our knowledge there is no evidence base for this recommendation. Further, data are conflicting as to whether placing the swab into a non-selective transport medium such as Amies or Stuarts transport medium, prior to inoculation into a selective enrichment broth, decreases the yield of GBS[7,8]. Moreover, selective enrichment broth, which enhances GBS growth, has been shown to increase GBS detection by up to 50%[9].

Other studies evaluating the usefulness of transport media have provided conflicting information in reliably defining GBS colonization rates. In a clinical-based study conducted in Rhode Island, USA, Silver and Struminsky[8] compared the collection of two swabs to determine the difference between direct inoculation into a selective Todd–Hewitt broth versus delayed inoculation from Stuart's transport medium. This study found that compared to direct inoculation onto selective solid medium, inoculation into selective broth without delay missed 4.6% of positive GBS culture results; with delay 16.3% and without selective broth 31.9% were missed[8].

Another clinically based study, performed in the USA by Crisp and co-workers found there was no statistically significant difference when direct inoculation into a selective medium was compared to delayed inoculation from a standard transport medium[9]. However, the time the swab remained in transport medium was minimal, up to 2 hours[9], and this is not necessarily a practical time period in clinical practice.

Whereas we at the Women's and Children's Health, Melbourne, Australia once had swabs inoculated directly into media at the bed side, the recent merger of the Microbiology services of the two teaching hospitals to one geographical site has meant re-evaluation of practices. Of particular concern were the longer transport times before inoculation into selective broth for optimal detection of GBS carriage in pregnant patients.

As there were no data published to determine whether placing swabs in transport media for greater than 2 hours reduced the isolation rate of GBS, we designed a study to address this, to assist in changes to our service requirements.

### MATERIALS AND METHODS

From stored clinical GBS isolates collected from routine antenatal screening over the previous 6–8 weeks, 103 were randomly chosen for *in vitro* testing. After retrieval of clinical cultures, the GBS isolates were grown on Horse Blood Agar (HBA) plates by overnight incubation at 35°C in air. Each isolate was then suspended in sterile Brain Heart Infusion (BHI) broth and diluted equivalent to a 0.5 McFarland standard to obtain a concentration of approximately $1 \times 10^8$ cfu/ml.

The prepared inoculum was then diluted using three 100-fold dilutions to achieve a final dilution of 100 cfu/ml (10 cfu/swab). This was performed by adding 25 μl of inoculated broth to 2.5 ml of sterile BHI for each dilution. A viable count of the final dilution was determined by culture of 100 μl of the dilution onto an HBA plate, in duplicate.

Direct inoculation to emulate direct bedside procedure was then performed by pipetting 100 μl of the final diluted GBS suspension in BHI onto a sterile rayon swab and this swab was then placed immediately into the NPC broth[1,3], (a selective enrichment broth consisting of a Todd–Hewitt base with selective agents, nalidixic acid, colistin and crystal violet added) and incubated for 18–24 hours, aerobically at 35°C. This was time zero. Also at time zero, 100 μl of the final suspension in BHI was inoculated onto four rayon swabs which were then placed into Amies transport medium with charcoal (Venturi Transystem™, Copan, Italy). Swabs were stored in the transport medium for 2, 4, 6 and 24 hours, after which time the swabs were removed from the transport medium and placed into NPC broth and incubated for 18–24 hours in air at 35°C.

The swabs from the incubated NPC broths were then removed and placed into New Granada Medium Modified (NGM)[10] and incubated for 18–24 hours in air at 35°C. The presence of GBS was detected by the growth of the organism and the production of the characteristic and specific orange pigmented colonies in the medium[10]. The growth was quantified as negative if there were no colonies, + if there were 1–10 colonies, and ++ if there were greater than 10 colonies. All negative NGMs were subcultured onto solid medium to confirm negative results.

**Table 1** Summary of GBS screening results of swabs from transport media to NGM at specified time intervals

| *Total no. of isolates* | *Viable count of inoculum on swab prior to placement into transport medium (cfu/ml)* | *Growth in NGM after time in transport medium* | | | | | |
|---|---|---|---|---|---|---|---|
| | | *0 hour* | *2 hours* | *4 hours* | *6 hours* | *24 hours* | *Notes* |
| 95 | 6–164 | +/++ | +/++ | +/++ | +/++ | +/++ | Positive in all tubes, all times |
| 4 | 10–32 | N | N/ +/++ | +/++ | +/++ | ++ | Positive by 2 or 4 hours |
| 2 | 12–18 | N | ++ | N | ++ | ++ | Inconsistent result number 1 |
| | | N | N | + | N | + | Inconsistent result number 2 |
| 2 | 8–26 | N | N/ $+^{(w)}$ | N | N | N/ $+^{(w)}$ | Non-hemolytic GBS strains |
| Total 103 | 6–164 | N (8%) | N (0%) $+^{(w)}$ (4%) | N (3%) | N (3%) | N (0%) $+^{(w)}$(2%) | |
| | | +/++ (92%) | +/++ (96%) | +/++ (97%) | +/++ (97%) | +/++ (98%) | |

N, no orange pigment observed in New Granada Medium Modified (NGM); $+^{(w)}$, weak orange pigment observed (not characteristic orange); +, 1–10 orange colonies observed in NGM; ++, > 10 orange colonies observed in NGM

### RESULTS

Of all the isolates evaluated, 92.2% (95/103) were positive in all tubes at all time intervals. The growth in these tubes ranged from + to ++ from time intervals of 0–24 hours. See Table 1 for a summary of results of the 103 isolates. At time zero, 3.9% (4/103), were negative but became positive at either 2 or 4 hours. All four tubes in this group had ++ growth observed at 24 hours. In addition, two isolates inoculated gave inconsistent results. Both of these results are listed in Table 1 and show tube growth changing from not detected, to + or ++ and again to not detected. Another two isolates were non-hemolytic strains and therefore had no characteristic orange pigment detected in NGM, (or very pale observed growth, not characteristic of the beta-hemolytic strains). This demonstrates the specificity of the detection medium for beta-hemolytic strains of GBS, as originally described in 1986[11].

### DISCUSSION

Consistently positive results were obtained regardless of the time delay intervals of 0, 2, 4, 6 and 24 hours and did not make a difference to the detection rate. In 2.9% of cases the detection rate improved with delayed inoculation of 2 or 4 hours. Another 1.9% of isolates used gave inconsistent results; these results are likely explained by the use of such a low and controlled inoculum size in the experiment. The non-hemolytic GBS strains (1.9%) would not have been detectable in the medium chosen (NGM), as it relies on pigmentation production, not made by non-hemolytic streptococci[10,11].

In contrast to Silver and co-workers[8] we did find a significant difference in detection of GBS after many hours in non-nutritive, non-selective transport medium. However, the results from this study could have been confounded by the study design whereby directly inoculated specimens were compared with those placed into transport media left in the clinic until the end of the day for a mean of $9.9 \pm 8.5$ hours, then plated onto three different agar media, prior to inoculation into selective growth medium. This would have diluted the original inoculum prior to going into selective medium. Similar to Crisp and co-workers[9] we report no loss of detectability of GBS when using enrichment broth after swabs have been in standard transport media. However, we were able to show that this was possible for up to 24 hours in the transport medium and not just up to 2 hours, as they reported[9].

In conclusion, even with such a low inoculum, passage of GBS through Amies transport medium with charcoal for up to 24 hours prior to inoculation into the selective enrichment broth maintains viability and with acceptable detection rates (98%). At Women's and Children's Health, the collection procedure change from direct inoculation into NGM to delayed inoculation into NPC broth and then NGM has been possible, without any decrease in GBS detection rates. The swabs in Amies transport medium with charcoal provide a good alternative, where selective enrichment broth cannot be directly inoculated.

### REFERENCES

1. Garland SM. Early onset neonatal group B streptococcus (GBS) infections: associated obstetric risk factors. *Aust NZ J Obstet Gynaecol* 1991;31:117–18
2. Early-onset group B streptococcus disease – United States, 1998–1999. *MMWR Morbid Mortal Wkly Rep* 2000;49:793–6
3. Garland SM, Fliegner JR. Group B streptococcus (GBS) and neonatal infections: the case for intrapartum chemoprophylaxis. *Aust NZ J Obstet Gynaecol* 1991;31:119–22
4. Garland SM, Kelly N. Early-onset neonatal group B streptococcal sepsis: economics of various prevention strategies. *Med J Aust* 1995;162:413–17
5. Philipson EH, Palermino DA, Robinson A. Enhanced antenatal detection of group B streptococcal colonization. *Obstet Gynecol* 1995;85:437–9
6. Prevention of Perinatal Group B Streptococcal Disease – Revised Guidelines from CDC. *Morbidity and Mortality Weekly Report*. Recommendations and Reports, Centre for Disease Control & Prevention (CDC), August 16, 2002, Volume 51, No. RR-11
7. Altaie SS, Dryja D. Detection of group B streptococcus: comparison of solid and liquid culture media with and without selective antibiotics. *Diagn Microbiol Infect Dis* 1994;18:141–4
8. Silver HM, Struminsky J. A comparison of the yield of positive antenatal group B streptococcus cultures with direct inoculation in selective growth medium versus primary inoculation in transport medium followed by delayed inoculation in selective growth medium. *Am J Obstet Gynecol* 1996;175:155–7
9. Crisp BJ, Yancey MK, Uyehara C, Nauschuetz WF. Effect of delayed inoculation of selective media in antenatal detection of group B streptococci. *Obstet Gynecol* 1998;92:923–5
10. Kelly VN, Garland SM. Evaluation of New Granada Medium (modified) for the antenatal screening of Group B streptococcus. *Pathology* 1994;26:487–9
11. Tapsall JW. Pigment production by Lancefield-group-B streptococci (*Streptococcus agalactiae*). *J Med Microbiol* 1986;21:75–81

Received 03/03/03; Accepted 07/16/03
